# The impact of tuberculosis co-infection on virological failure among adults living with HIV in Ethiopia: A systematic review and *meta*-analysis

**DOI:** 10.1016/j.jctube.2022.100310

**Published:** 2022-03-04

**Authors:** Temesgen Getaneh, Ayenew Negesse, Getenet Dessie, Melaku Desta

**Affiliations:** a Department of Midwifery, College of Health Science, Debre Markos University, P.O BOX 269, Debre Markos, Ethiopia; b Department of Human Nutrition and Food Science, College of Health Science, Debre Markos University, Debre Markos, Ethiopia; c Center of excellence in Human Nutrition, School of Human Nutrition, Food Science and Technology, Hawassa University, Ethiopia; d Department of Nursing, College of Health Science, Bahir Dar University, Bahir Dar, Ethiopia

**Keywords:** HIV, TB, Virological unsuppressed, Systematic review, Meta-analysis, Ethiopia

## Abstract

**Introduction:**

Tuberculosis (TB) is the most common serious opportunistic infection among people with Human Immunodeficiency Virus (HIV) infection and are considered as the double burden diseases of the world. TB is the leading cause of death among people living with HIV, accounting one in three HIV related deaths. Although TB is responsible for high burden of virological unsuppressed in Ethiopia, there is no national level evidence. Therefore, this systematic review and *meta*-analysis was aimed at estimating the pooled burden of virological unsuppressed among adults with both HIV-TB and impact of TB on virological failure in Ethiopia.

**Methods:**

The finding of this *meta*-analysis was reported using the Preferred Reporting Items for Systematic Reviews and Meta-Analyses checklists. Major data bases PubMed, Scopus, Cochrane Library, Science Direct and Google scholar were searched to access articles. Cochran’s Q statistic quantified with inverse variance was computed to check heterogeneity. Funnel plot visualization and Egger’s test were fitted to assess publication bias across included studies. Random effects model *meta*-analysis using STATA version-15 statistical software was used to estimate the pooled effect with respective 95% confidence intervals.

**Results:**

A total of 15 primary studies reporting on impact of tuberculosis on virological unsuppressed among adults living with HIV in Ethiopia were eligible for this *meta*-analysis. Accordingly, the pooled prevalence of virological unsuppressed among adults with both HIV-TB in Ethiopia was 39.09% (95% CI: 29.04, 49.15). In addition, the odds of virological unsuppressed among adults with both HIV-TB was 2.46 times higher when compared with adults living with HIV infection alone (OR = 2.46, 95% CI: 1.74, 3.46).

**Conclusion:**

The present systematic review and *meta*-analysis evidenced that the pooled prevalence of virological unsuppressed among adults with both HIV-TB was much higher than virological unsuppressed among adults living with HIV alone. Moreover, the odds of virological failure among adults with both HIV-TB was significantly higher when compared with among only HIV infection in Ethiopia. Therefore, strengthening TB prevention interventions, early identification and managing the case and prioritizing viral load monitoring and adherence support among adults living with HIV are recommended.

## 1 Introduction

**Fig. 1:**
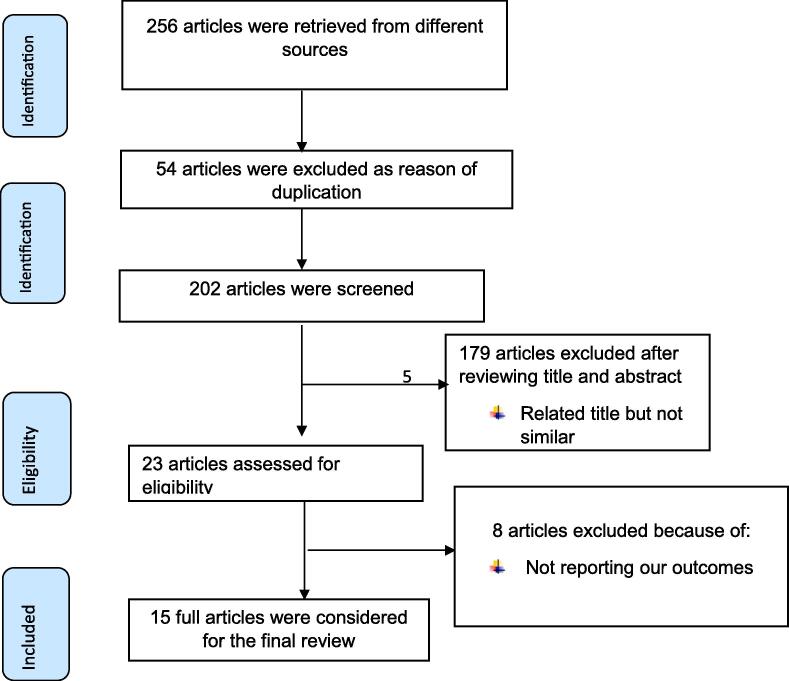
PRISMA flow diagram of included studies to estimate the pooled impact of TB co-infection on virological failure among adults living with HIV in Ethiopia.

**Fig. 2:**
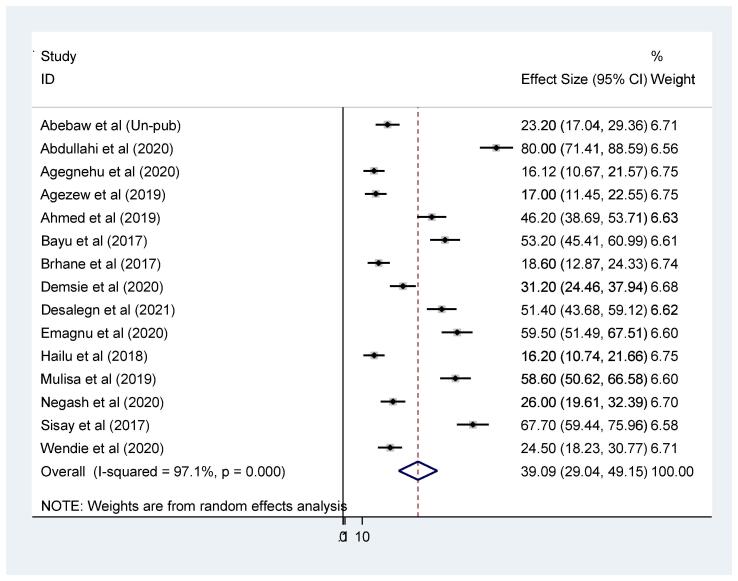
Forest plot of the pooled prevalence of virological failure among adult patients living with HIV-TB co-infection in Ethiopia.

**Fig. 3:**
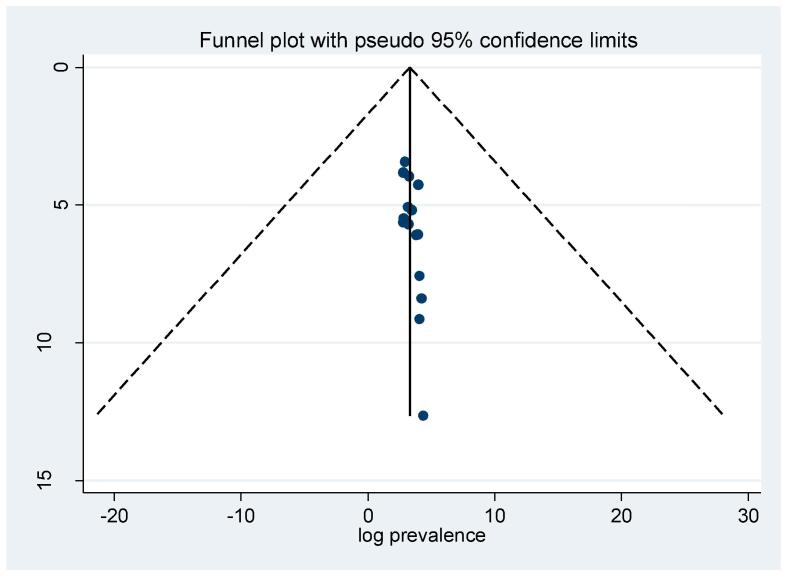
Meta funnel presentation of the pooled prevalence of virological failure among adult patients living with HIV-TB co-infection in Ethiopia.

**Fig. 4:**
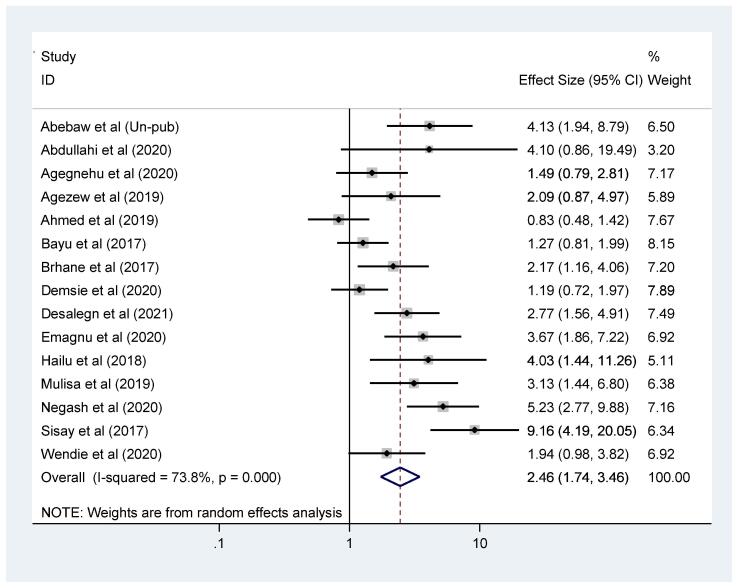
Forest plot of the impact of TB co-infection on virological failure among adults living with HIV in Ethiopia.

HIV continues to be a major global public health issue, having claimed 36.3 million lives so far[1]. Increasing access to effective HIV prevention, and treatment including for opportunistic infections, HIV infection has become a manageable chronic health condition, enabling people to lead long and healthy lives even though there is no cure for HIV infection [2], [3]. There were an estimated 37.7 million people living with HIV at the end of 2020, over two thirds of whom (25.4 million) are in the World Health Organization African Regions. In 2020, 680,000 people died from HIV-related causes and 1.5 million people acquired HIV[1].Fig. 1.Fig. 2.Fig. 3.Fig. 4.Table 1.Table 2.Table 3..

**Table 1:** Descriptive summary of 15 studies included in the systematic review and *meta*-analysis of impact of TB co-infection on virological failure among adults living with HIV in Ethiopia.

Author	Pub. Year	Region	Study design	Sampling technique	Sample size	Prevalence (%)	OR	JBI
Abebaw et al[24]	Un-pub	Amhara	cross–sectional	Simple random	69	23.2	4.1	Low risk
Abdullahi et al[19]	2020	Oromo	case-control	Simple random	10	80	4.1	Low risk
Agegnehu et al[25]	2020	Amhara	Retrospective Cohort	Simple random	93	16.12	1.5	Low risk
Agezew et al[26]	2019	Amhara	Retrospective Cohort	Systematic	47	17	2.0	Low risk
Ahmed et al[27]	2019	Amhara	case-control	Systematic	67	46.2	0.8	Low risk
Bayu et al[28]	2017	Amhara	case-control	Simple random	137	53.2	1.2	Low risk
Brhane et al[29]	2017	Amhara	cross–sectional	Systematic	129	18.6	2.1	Low risk
Demsie et al[30]	2020	Tigray	cross–sectional	Simple random	80	31.2	1.2	Low risk
Desalegn et al[31]	2021	Oromo	case-control	Systematic	68	51.4	2.7	Low risk
Emagnu et al[32]	2020	Amhara	case-control	Simple random	42	59.5	3.6	Low risk
Hailu et al[18]	2018	Tigray	Retrospective Cohort	Consecutive	43	16.2	4	Low risk
Mulisa et al[33]	2019	Oromo	case-control	Systematic	29	58.6	3.1	Low risk
Negash et al[34]	2020	Tigray	cross–sectional	Consecutive	123	26	5.2	Low risk
Sisay et al[35]	2017	Addis Ababa	Retrospective Cohort	Simple random	31	67.7	9.1	Low risk
Wendie et al[36]	2020	Amhara	Retrospective Cohort	Simple random	57	24.5	1.9	Low risk

**Table 2:** Sub group analysis which describes pooled impact of TB co-infection on virological failure among adults living with HIV in Ethiopia.

Subgroup	Categories	No of studies	prevalence (95%CI)	Heterogeneity statistics	I^2^	p-value
Region	Amhara	8	32.07(20,43)	169.38	95.9	<0.001
	Addis Ababa	1	67.7(59,75)	0.0	–	---
	Tigray	3	24.3(15,33)	12.46	83.9	0.002
	Oromo	3	63.2(46,79)	24.8	91.9	0.004
Study design	Cross sectional	4	24.5(19,29)	8.22	63.5	0.042
	Cohort	5	28.0(12,43)	129.2	96.9	<0.001
	Case control	6	58.0(49,67)	38.92	87.2	0.010
Sampling technique	Consecutive	2	20.9(11,30)	5.23	80.9	0.022
	Systematic sampling	5	38.1(21,55)	289.6	96.9	<0.001
	Simple random	8	44.2(28,60)	50.95	97.6	<0.001

**Table 3:** for the included studies to identify source of heterogeneity for the pooled prevalence of virological failure among adult patients living with HIV-TB co-infection in Ethiopia.

**Variables**	**Coefficients**	**p-value**
Study year	−0.255	0.986
Sample size	−0.762	0.621
Study design		
Retrospective cohort	−1.822	0.843
Case control	31.64	0.657
Cross sectional	Reference	Reference
Region		
Amhara	−35.96	0.654
Oromo	−6.31	0.751
Tigray	−43.15	0.431
Addis Ababa	R**eference**	R**eference**

Worldwide, tuberculosis is the most common serious opportunistic infection among people with HIV infection and are considered as the double burden diseases of the world [3]. TB is the leading cause of death among people living with HIV, accounting one in three Acquired Immunodeficiency syndrome related deaths. According to the recent report, 10 million people fill ill with TB and 1.6 million died from the disease, of which 26% were due to HIV and TB co-infection [4]. In 2018, 251,000 people died from HIV-TB co-infection [5]. From low and middle income countries, African continent takes the greater share (74%) of the 1.2 million HIV-TB cases worldwide due to the burden of HIV [4], [6]. In Ethiopia, 25.6% of HIV patients were co-infected with TB [7], [8].

TB and HIV co-infections place an immense burden on health care systems and pose particular diagnostic and therapeutic challenges [9]. First, the management of HIV infections in persons with TB is complicated by several factors, including drug interaction, overlapping drug toxicities, exacerbation of side effects, concerns about adherence, and immune reconstitution inflammatory syndrome [10], [11], [12]. Next, even though the success rates of anti-retro viral therapy are considered as high, factors like TB, the most common opportunistic infection among HIV patients, is a significant predictors of virological unsuppressed [13]. Scholars suggest that virological unsuppressed is more likely to ensue in patients diagnosed with TB [14], [15]. This results a high incidence and tuberculosis-related mortality among patients with virological unsuppressed [16]. In addition, virological unsuppressed of first line regimens creates the need for very expensive and difficult to implement second line regimens which are often unaffordable and largely donor dependent in resource limited settings [17].

HIV associated TB presents a risk to achieving the Sustainable Development Goals particularly in low and middle income countries. To reach the new proposed global 95–95–95 targets, it is required to redouble our efforts to avoid the worst-case scenario of a half million excess HIV-related deaths in sub-Saharan Africa, due to the slowing public health response to HIV.

In Ethiopia, the burden of virological unsuppressed among adults with both HIV-TB ranges from 16% [18] to 80% [19]. Although TB-HIV co-infection is a major public health problem and responsible for high burden of virological unsuppressed in Ethiopia, there is no national level evidence on impact of HIV-TB co-infection on virological unsuppressed among adult patients. Therefore, this systematic review and *meta*-analysis was aimed at estimating the pooled virological unsuppressed among adults with both HIV-TB and impact of TB on virological unsuppressed in Ethiopia using inconsistent available researches.

## 2 Methods

### 2.1 Searching strategy and reporting

In general, this systematic review and *meta*-analysis was targeted at exploring pooled burden of virological unsuppressed among adults with both HIV-TB and impact of TB infection on virological unsuppressed in Ethiopia. The finding of this *meta*-analysis was reported using the Preferred Reporting Items for Systematic Reviews and Meta-Analyses (PRISMA) checklist [20] (Additional file-1). Major electronic data bases including PubMed, Scopus, Cochrane Library, Science Direct and Google scholar were searched to access articles. Local institutional online repositories and the reference list of already identified articles were also searched. Search terms (“Virological failure”, “virological unsuppressed”, “Treatment failure”, “Treatment outcome”, “Tuberculosis-human immunodeficiency virus co-infection”, “Opportunistic infection”, “Human immunodeficiency virus”, “Acquired immunodeficiency syndrome”, “Adult Patients” and “Ethiopia”) combined with Boolean operators (“AND” and “OR”) were applied. The overall comprehensive searching was conducted from September 20/2021 to October 9/2021.

## 3 Inclusion and exclusion criteria

Observational studies including cross sectional studies, case control studies and cohort studies reporting either the burden of virological unsuppressed among adults with both HIV-TB or the impact of TB on virological unsuppressed among adults living with HIV in Ethiopia were included. A total of 15 studies (14 published articles and one unpublished study) written in English language were analyzed to estimate the pooled impact of HIV-TB co-infection on virological unsuppressed. No restriction was applied on publication status, year of study, and study setting. Finally, studies unable to access full texts (after two times email request was attempted to the primary authors), and studies scoring below 6 using JBI critical appraisal criteria were excluded in this met-analysis.

### 3.1 Quality assessment

After duplicated studies were removed using EndNote version-7.2 citation manager, two independent reviewers evaluated all searched primary studies. Joanna Briggs Institute (JBI) critical appraisal tool adapted for observational studies (cross sectional, case control and cohort) was utilized to assess the quality of primary studies. JBI criteria applied for evaluating cross sectional studies have 8 criteria, 10 for case control studies and 11 criterias for cohort studies. Any disagreement between reviewer was solved with discussion and census; if not third reviewer was involved. Finally, studies scoring half and above included in the final *meta*-analysis of this study.

### 3.2 Data extraction and outcome of measurement

A standard Excel spreadsheet format developed based on Joanna Briggs Institute Reviewers’ Manual [21] was utilized for data extraction. The format includes: first author name, study area, publication year,‘ study design, sampling technique, sample size, prevalence of virological failure among HIV-TB co-infection and cross tabulation to analyze the impact of TB co-infection on adult living with HIV virological unsuppressed.

**Virological failure/** unsuppressed: adults living with HIV whose plasma viral load of ≥ 1,000 copies/mL in two consecutive viral load measurements in a 3-month interval after 6 months of starting a new anti-retro viral regimen were considered as virological failure/ unsuppressed.

### 3.3 Statistical analysis

Data extracted on excel spread sheet were imported to STATA version 14 for the main analysis. Then, random effect model *meta*-analysis was utilized to report the pooled prevalence of virological unsuppressed among adults with both HIV-TB. Forest plot with corresponding 95% confidence interval was used to present the result. Cochran’s Q statistic with inverse variance (I^2^) was used to assess the existence of statistical heterogeneity and to quantify it. Low, moderate and high heterogeneity were considered at 25%, 50% and 75% respectively [22]. Publication bias was also assessed using Egger’s regression test [23] and funnel asymmetry plot. The presence of publication bias was declared when p value<0.05. In addition, subgroup analysis was also computed to explore those potential sources of heterogeneity across primary studies.

## 4 Results

### 4.1 Search results and characteristics of included studies

At first, 256 primary studies were retrieved from major data bases. Then, 54 studies were removed due to duplication. After reviewing titles and abstracts of 202 studies, 179 articles were excluded (related title but not similar). Among 23 remaining studies, eight studies were excluded because their outcomes not related with our objectives. Finally, 15 primary studies were included to evaluate the impact of TB co-infection on virological unsuppressed among adults living with HIV in Ethiopia (Fig-1).

A total of 15 studies [18], [19], [24], [25], [26], [27], [28], [29], [30], [31], [32], [33], [34], [35], [36] and 1025 adults with both HIV-TB were included to estimate the pooled burden of virological unsuppressed among this patients. More than half of included studies (8) were from Amhara [24], [25], [26], [27], [28], [29], [32], [36]. Among the remaining articles, three were from Oromo [19], [31], [33], three from Tigray [18], [30], [34] and the remaining single article was from Addis Ababa [35]. Six of included primary articles were case control studies and the remaining five and four articles were reported using retrospective cohort and cross sectional design respectively. In addition, the lowest prevalence of virological unsuppressed among adults with both HIV-TB were 16.1% reported in Amhara while the highest was 80% which was reported in Oromo. Regarding to JBI quality assessment, each primary studies were appraised using their respective checklists and all 15 articles scored more than 50% (low risk) (Table-1).

### 4.2 Meta-analysis

According to the *meta*-analysis 15 primary studies, the pooled prevalence of virological unsuppressed among adults with both HIV-TB in Ethiopia was 39.09% (95% CI: 29.04, 49.15). However, the forest plot indicated that substantial heterogeneity across primary studies was observed (I^2^ = 97.1%) (Fig-2). Therefore, random effect model *meta*-analysis was appropriate to pool the effect. As a result, subgroup analysis using study region, study design and sampling technique was conducted to explore potential source of heterogeneity. Egger’s statistical test and asymmetrical funnel plot test were applied to check publication bias across included studies. In regarding, non-significant Egger’s test (p = 0.204) and relatively symmetrical visualization of funnel plot (Fig-3) exclude publication bias across included studies.

Regarding to individual studies weight, three individual studies [18], [25], [26] have higher weight effect which might implies the studies have larger sample size, or repeated several times, and has narrower confidence interval, good estimation. While one study have the lowest weight effect [19] which might indicated small sample size, wide confidence interval and poor estimation (Figure-2).

### 4.3 Subgroup analysis

Regarding to subgroup analysis, higher burden of virological unsuppressed among adults living with HIV-TB co-infection was observed in capital city of Ethiopia (Addis Ababa) and Oromia regional state. Whereas, lower prevalence of virological unsuppressed among adults living with HIV-TB co-infection was detected in Amhara Tigray regional states. In addition, higher case report of virological unsuppressed among adults living with HIV-TB co-infection was evidenced among studies conducted using case control design (Table-2).

### 4.4 Meta regression

In order to identify possible source of heterogeneity of the pooled prevalence of of of virological unsuppressed among adults living with HIV/TB in Ethiopia. This *meta*-eta-regression was undertaken by considering both continuous and categorical data. Sample size, study year, setting and study region for each individual studies were considered in the *meta*-regression. But, the *meta*-regression showed that the pooled prevalence of pooled prevalence of virological failure among adult patients living with HIV-TB co-infection in Ethiopia.

### 4.5 Impact of TB on virological failure

In addition, this *meta*-analysis also showed that virological unsuppressed was significantly increased among adult patients living with HIV-TB co-infection. Thus, the odds of virological unsuppressed among adults with both HIV-TB was 2.46 times higher when compared with adults living with HIV infection alone (OR = 2.46, 95% CI: 1.74, 3.46) (Fig-4). Furthermore, the *meta*-analysis of cohort and case control studies (after exclusion of cross sectional studies to check cause-effect relationship), the odds of virological unsuppressed among adults with both HIV-TB was 2.39 times higher than patients who had only HIV infection (OR = 2.39; 95% CI: 1.58, 3.60).

Moreover, a study conducted in Amhara [28] have the largest weight effect indicating a good estimation and narrow confidence interval. Whereas a study conducted in Oromo [19] reported the lowest weight estimation in turn indicate small sample size, wide confidence interval and poor estimation (figure-4).

## 5 Discussion

Generally, the present systematic review and *meta*-analysis was aimed at estimating the pooled burden of virological unsuppressed among adults living with HIV-TB co-infection. It also stand at exploring the pooled effect of TB infection on virological unsuppressed among Ethiopian adults living with HIV. The pooled prevalence of virological unsuppressed among adults living with HIV-TB co-infection in Ethiopia was 39.09% (95% CI: 29.04, 49.15) even though substantial heterogeneity across primary studies detected. This finding is much higher than virological unsuppressed among general population in Ethiopia which was 5.6%. Tuberculosis enhances progression of HIV infection. Different evidence confirmed that the recovery of CD_4_ + T-cells among patients who had HIV-TB co-infection was poor. In addition, in HIV-TB patients sever CD_4_ + T-cells lymphocytopenia and impaired immune restoration were detected [37], [38]. Moreover, decreased adherence to HIV treatment during TB treatment because of high pill burden and side-effects might enhance viral replication [39]. All this issues will contribute to HIV treatment unsuppressed among patients which could be detected using viral load (gold standard). It is also higher than finding of studies reported in South Africa [40] and Uganda [41]. This could be due to difference in socio-economic, health service coverage, burden of TB-HIV co-infection and treatment coverage.

Countries like Ethiopia with a high burden of HIV-associated TB need to rapidly integrate and scale up their TB/HIV services. Early and frequent TB screening and testing for people newly diagnosed with HIV is essential and the delay between diagnosis and treatment must be drastically reduced. HIV and TB services require coordination of multisector efforts to find the missing millions due to virological unsuppressed and its consequences. Scholars showed that the incidence and TB-related mortality was higher among patients with virological unsuppressed[16], [42]. Informing and engaging populations about their increased risk of TB and HIV and facilitating better access to client-centered TB and HIV prevention, diagnostic and treatment services, and integrating TB and HIV service delivery were needed [43].

Regarding to subgroup analysis, higher burden of virological unsuppressed among adults living with HIV-TB co-infection was observed in capital city of Ethiopia (Addis Ababa) and Oromia regional state. Whereas, lower prevalence of virological unsuppressed among adults living with HIV-TB co-infection was detected in Amhara Tigray regional states. The difference across regional states could be due to difference in HIV and TB prevalence, detection of viral load or diagnosis of virological unsuppressed and prevention of TB. In addition, variation of health care service utilization including HIV and TB treatment and support service, accessibility of medications and services on demand might be responsible for this variation.

In addition, the odds of virological unsuppressed was significantly higher among adults living with HIV-TB co-infection when compared with adults living with HIV only. This finding is in line with studies conducted in Uganda and South Africa [44], [45]. Findings from Tanzania [46] and India [14] also supported the present *meta*-analysis result. TB and HIV, potentiate one another, accelerating the deterioration of immunological functions. In high-burden settings like Ethiopia, HIV coinfection is the most important risk factor for developing active TB [7]. TB infection also has a negative impact on the immune response to HIV, accelerating the progression from HIV infection to AIDS in turn leads to long lasting immune suppression and increase in viral load or treatment failure [47], [48]. TB often is an early HIV opportunistic infection, it may particularly favour early viral replication and dissemination, and therefore contribute to progression of HIV disease [49]. Prevention of TB and early detection of virological failure and adoption of appropriate measures to ensure viral suppression and immune recovery are very important to reduce HIV related morbidity and mortality.

Even though this *meta*-analysis is the first to be conducted in Ethiopia to estimate pooled effect of TB co-infection on virological failure among adults living with HIV, it is not without limitation. First, the eligible studies were only from four regional state of Ethiopia (didn’t include all regional state of the country). Secondly, substantial heterogeneity among primary studies included to estimate effect size was observed although subgroup analysis was reported. Thirdly, this systematic review include cross sectional studies in turn difficult to predict the cause-effect relationship (even if cause-effect was checked after cross sectional studies excluded).

## 6 Conclusion

The present systematic review and *meta*-analysis evidenced that the pooled prevalence of virological failure among adults living with HIV-TB co-infection was much higher than virological failure among adults living with HIV alone. Moreover, the odds of virological failure among adults living with HIV-TB co-infection was significantly higher when compared with among only HIV infection in Ethiopia. Therefore, strengthening TB prevention interventions, early identification and managing the case and prioritizing viral load monitoring and adherence support among adults living with HIV are recommended.

## Declaration of Competing Interest

The authors declare that they have no known competing financial interests or personal relationships that could have appeared to influence the work reported in this paper.
